# Transcriptomic era of cancers in females: new epigenetic perspectives and therapeutic prospects

**DOI:** 10.3389/fonc.2024.1464125

**Published:** 2024-11-13

**Authors:** Runhe Zhu, Jiawei Ni, Jiayin Ren, Dongye Li, Jiawei Xu, Xinru Yu, Ying Jie Ma, Luan Kou

**Affiliations:** ^1^ The Traditional Chinese Medicine College, Shandong University of Traditional Chinese Medicine, Jinan, China; ^2^ The Pharmacy College, Shandong University of Traditional Chinese Medicine, Jinan, China; ^3^ The First Clinical College, Shandong University of Traditional Chinese Medicine, Jinan, China; ^4^ Shandong Provincial Maternal and Child Health Care Hospital Affiliated to Qingdao University, Jinan, China

**Keywords:** transcriptomic, era, cancers, females, epigenetic

## Abstract

In the era of transcriptomics, the role of epigenetics in the study of cancers in females has gained increasing recognition. This article explores the impact of epigenetic modifications, such as DNA methylation, histone modification, and non-coding RNA, on cancers in females, including breast, cervical, and ovarian cancers (1). Our findings suggest that these epigenetic markers not only influence tumor onset, progression, and metastasis but also present novel targets for therapeutic intervention. Detailed analyses of DNA methylation patterns have revealed aberrant events in cancer cells, particularly promoter region hypermethylation, which may lead to silencing of tumor suppressor genes. Furthermore, we examined the complex roles of histone modifications and long non-coding RNAs in regulating the expression of cancer-related genes, thereby providing a scientific basis for developing targeted epigenetic therapies. Our research emphasizes the importance of understanding the functions and mechanisms of epigenetics in cancers in females to develop effective treatment strategies. Future therapeutic approaches may include drugs targeting specific epigenetic markers, which could not only improve therapeutic outcomes but also enhance patient survival and quality of life. Through these efforts, we aim to offer new perspectives and hope for the prevention, diagnosis, and treatment of cancers in females.

## Introduction

Disease occurrence results from the gradual accumulation of changes that affect the structure and function of the genome. Genetic alterations directly disrupt Deoxyribonucleic Acid (DNA) sequences and interfere with the normal functions of genes. In contrast, epigenetics contributes to cancer formation by regulating gene expression programs that promote tumor development. Although these epigenetic changes do not alter the DNA sequence directly, they can facilitate the acquisition of hallmark features of cancer by influencing gene expression (2).However, unlike the irreversibility of genetic changes, the reversibility of epigenetic modifications makes them particularly appealing for drug development and clinical treatments. This reversibility allows epigenetic modifications to be dynamically regulated through drug interventions, offering more flexibility and potential intervention points for developing treatment strategies. Therefore, targeting epigenetic mechanisms not only opens new avenues for precision medicine but also plays a significant role in treating various diseases. Additionally, epigenetic factors can control key biological processes such as cell differentiation and embryogenesis by fine-tuning gene expression programs, thereby playing a critical role in regulating these processes. Furthermore, compelling evidence suggests that epigenetic reprogramming is closely linked to dynamic transcriptomic heterogeneity in cancer, reinforcing its role as a cancer-driving factor (3). Among the various epigenetic markers studied in humans, DNA methylation has garnered the most attention from scientists (4). Extensive research has convincingly demonstrated that changes in DNA methylation patterns play a crucial role in coordinating tumor development and metastasis. These studies revealed how DNA methylation influences tumor progression and spread by regulating gene expression patterns, offering important insights into the biological mechanisms underlying cancer (5). In addition to alterations in DNA methylation patterns, cancer often involves abnormal histone and RNA modifications. These epigenetic changes not only occur independently but also interact with each other, severely disrupting the cell’s transcriptome. This disruption further undermines cellular homeostasis, impairs normal cell functions, and disturbs biological processes, thereby creating a conducive environment for cancer development and progression (6). These findings have provided new insights into treating tumors in females, which can significantly enhance the application of traditional pathology in clinical management and improve patient prognosis. Once the epigenetic characteristics of human tumors are identified, these markers will open new avenues for precise diagnosis and may even help define new tumor subtypes. Moreover, they can be employed for recurrence detection, monitoring residual lesions, and guiding treatment decisions, thereby offering valuable information and support for personalized medicine (2).

### Current status and importance of cancers in females

Cancers in females, including the prevalent types such as breast, cervical, and ovarian cancer, pose a significant health threat to women and impose substantial economic and psychological burdens on families and societies. Breast cancer is one of the leading causes of death among postmenopausal women, accounting for approximately 23% of all cancer-related deaths, making it a global challenge (7). Unfortunately, due to a lack of awareness regarding self-examinations and the importance of regular clinical screenings, breast cancer is often diagnosed at an advanced stage.

In 1990, the global average mortality rate of breast cancer was estimated at 13.77 per 100,000 people. Between 1990 and 2015, the mortality rate increased annually by 0.7 per 100,000 people (8). In 2020, breast cancer claimed the lives of 685,000 women, accounting for 16% of all female cancer deaths, meaning that 1 in every 6 women who died of cancer succumbed to breast cancer. Since 2007, several high-income countries in North America, Europe, and Oceania have reported a continuous increase in the incidence of both premenopausal and postmenopausal breast cancers (9). In China, the cancer-related health burden continues to grow, with over 16 million people diagnosed with cancer annually and 12 million dying from it. Similar to most countries, breast cancer has become the most common cancer among Chinese women. In 2014, China accounted for 12.2% of all newly diagnosed breast cancer cases worldwide, and breast cancer deaths in China accounted for 9.6% of the global total (10). In 2020, 416,371 Chinese women were diagnosed with breast cancer, accounting for 18% of all new breast cancer cases worldwide (11). It is projected that by 2040, the number of newly diagnosed breast cancer cases will increase by over 40%, reaching approximately 3 million cases annually. Simultaneously, the number of deaths from breast cancer is expected to increase by more than 50%, from 685,000 in 2020 to 1 million in 2040 (12). There is significant heterogeneity in the patterns of post-traumatic responses among breast cancer patients, with those undergoing treatment exhibiting varying degrees of post-traumatic stress symptoms and growth (13). Aerobic and mind-body exercises and comprehensive workout routines have been found to effectively improve the quality of life of women with breast cancer (14).

Cervical cancer is the third most common cancer among women globally, following breast and colorectal cancers, with approximately 569,000 new cases diagnosed annually (15). Epigenetic changes play a significant role in the development and metastasis of cervical cancer. In developing countries, cervical cancer is the most common type of cancer in women, accounting for approximately 25% of all cases of cancers in females (16). It is also the second leading cause of cancer-related death among women worldwide.

Approximately 570,000 new cases of cervical cancer were diagnosed in 2018, resulting in 311,000 deaths (17). By 2020, the number of new cervical cancer cases had risen to 604,127, with 341,831 deaths, accounting for 6.5% and 7.7% of all new cancer cases and deaths among women worldwide, respectively (18). Among the 31 countries analyzed, the majority (26 in the incidence analysis and 30 in the mortality analysis) experienced stable or declining incidence and mortality rates over the past decade (19). In Vietnam, cervical cancer is the seventh most common type of cancer among women and the most prevalent gynecological cancer, with an incidence rate of 7.1 per 100,000 people. Both incidence and mortality rates in Vietnam depicted a slight decline from 1999 to 2017. In South Korea, a significant decrease in the incidence has been reported, highlighting the effectiveness of cervical cancer prevention and control programs in reducing the incidence of the disease. However, in Japan, the incidence and mortality rates of cervical cancer among women of all ages are on the rise, and this trend is expected to continue in the absence of effective prevention and intervention measures (20). From 1990 to 2019, the overall risk of cervical cancer incidence in the Chinese population gradually increased, with the age of the high-incidence groups advancing, while the risk of death decreased. It is anticipated that the incidence rate will decrease in the coming year, although the mortality rate is expected to rise before eventually falling (21). Due to its large population, China accounted for 11.9% of cervical cancer deaths and 12.3% of the global incidence rate in 2017 (22). Cervical cancer can significantly affect the psychological health of patients. Anticipatory grief, which is closely related to the impact of cancer and the patient’s physical condition, may also be influenced by factors such as the nature of the event, the patient’s age, and their physical manifestations (23).

Ovarian cancer accounts for 3% of all cancers in females but is the fifth leading cause of cancer death among women, following lung, breast, colorectal, and pancreatic cancers. In 2016, there were an estimated 57,200 new cases of ovarian cancer and 27,200 deaths in China (24). In 2018, the United States reported approximately 22,240 new cases and 14,070 deaths from ovarian cancer (25). By 2020, ovarian cancer was responsible for 207,252 deaths worldwide. The disease is categorized into various subtypes, with serous carcinoma being the predominant histological subtype, accounting for 42.97% of all new ovarian cancer cases worldwide (26). High-quality population-based cancer registry data will enhance our understanding of the epidemiological characteristics of ovarian cancer and provide valuable insights into its prevention, screening, and treatment.

The incidence of ovarian cancer increases with age and is particularly high among women aged more than 50 years, especially postmenopausal women. This heightened risk is associated with cessation of ovulation and related physiological changes. Although ovarian cancer is more common in older women, it can occur at any age. Factors such as genetics, environment, and lifestyle also contribute to its development (27).

### History and development of epigenetics in oncology

Epigenetics has become increasingly integral to oncology, evolving into a crucial aspect of cancer research. Early studies revealed that cancer development is driven by cumulative changes that affect genomic structure and function. While genetic mutations disrupt DNA sequences, epigenetic changes modulate gene expression programs that contribute to the acquisition of cancer hallmarks. DNA methylation and histone modifications are the primary epigenetic alterations observed (28). Transcriptional epigenetic regulation can be achieved through changes in DNA methylation, histone modifications, and chromatin remodeling. Growing evidence indicates that epigenetic dysregulation is a prevalent mechanism in cancer (29). Epigenetic abnormalities are recognized as markers of cancer onset and progression. Consequently, combination therapies that integrate epigenetic drugs or targets with immunotherapy can enhance antitumor immunity as an improved strategy for cancer management (30). Additionally, the close relationship between metabolism and epigenetics has been highlighted, suggesting that targeting these interactions may offer promising therapeutic strategies (31).

Consequently, epigenetics has become pivotal in modern cancer treatment strategies. Over the past decade, immunotherapy has emerged as the primary cancer treatment method. Despite its promise, most patients do not achieve complete recovery and may develop resistance to treatment. Addressing this challenge has become a major focus in oncological epigenetics. Recent studies have exhibited that the epigenetic characteristics of immune and cancer cells can serve as accurate predictors of response to immunotherapy. Furthermore, combining epigenetic drugs with immunotherapy has the potential to modulate responses to these treatments. This has led to the development of epigenetic combination therapies as a new direction for oncological research. Emerging evidence suggests that tumors often evade immune responses through various epigenetic mechanisms. Consequently, pharmacological modulation of epigenetic regulators could normalize impaired immune surveillance and trigger antitumor immune responses, offering new strategies for improving cancer treatment outcomes.

Molecular biomarkers commonly used to predict cancer responses to immunotherapy include Programmed Death-ligand 1 (PD-L1) expression (32), tumor-associated antigens (33), Human Leukocyte Antigen (HLA) expression (34), T Cell Receptor (TCR) repertoire assessment (35), tumor mutational burden and neoantigen identification (36), mismatch repair deficiencies, presence of tumor-infiltrating lymphocytes, and cells within the tumor microenvironment that may inhibit antitumor immune responses (37). Recent advances in cervical cancer biology have revealed that epigenetic changes are prevalent during the transformation and metastasis of the disease. Abnormal DNA methylation and histone modifications have been extensively studied in cervical cancer (38). Beyond cervical cancer, epigenetics also play a significant role in other cancers, such as lung cancer, which is characterized by well-defined genetic driver mutations and both global and locus-specific epigenetic modifications. Advances in molecular targeted therapy have transformed the treatment of gene-driven lung adenocarcinoma, although these cancers display genetically distinct subclones and exhibit phenotypic variability. While targeted therapies can produce rapid responses, changes in the epigenome can explain the heterogeneity in the initial responses and subsequent resistance to these treatments (39). In both hematologic malignancies and solid tumors, existing drugs often perform poorly against solid tumors, limiting their broader application. Therefore, it is crucial to uncover the mechanisms underlying resistance or insensitivity. Epigenetic tools may induce specific metabolic vulnerabilities in solid tumors, offering new opportunities for developing innovative combination treatment strategies (31). In hematologic malignancies, Branched-chain Amino Acid Transaminase 1 inhibitors have been found to effectively inhibit the proliferation of Enhancer Of Zeste Homolog 2-deficient leukemia-initiating cells (EZH2-deficient leukemia-initiating cells), both *in vitro* and *in vivo*. This inhibition is selective, sparing normal hematopoietic stem cells and hematopoietic processes. Moreover, inhibiting this metabolic pathway may also hold therapeutic potential for other hematologic malignancies with EZH2 mutations or dysregulation (40).

## Fundamental principles of epigenetics

### Epigenetic mechanisms

Epigenetic modifications refer to heritable yet reversible changes in gene expression that do not involve alterations in the DNA sequence. These modifications are crucial for regulating gene expression and are essential for controlling vital biological processes such as cell differentiation and embryonic development. In cancer, epigenetic reprogramming contributes to transcriptomic heterogeneity and significantly influences tumor development and progression. Unlike the slow process of genomic evolution, epigenetic changes occur rapidly, making them particularly prevalent in cancer cells. This rapid adaptability enables cancer cells to quickly adjust to environmental changes and develop mechanisms to evade immune surveillance and drug treatment. Therefore, a comprehensive study of epigenetic modifications is essential for understanding the mechanisms of cancer onset and progression as well as for developing more effective treatment strategies (**Table 1**).

**Table 1:** The table catalogs several prevalent malignancies in females, including ovarian and breast cancer, delineating their epigenetic alterations, and encapsulating the correlation between female tumors and epigenetic mechanisms.

Disease	Epigenetic changes	Clinical Drug Trials and Their Significance	Reference
Endometrial cancer	Nucleolar Protein 6 (NOL6) regulates the expression of Twist-related Protein 1 (TWIST1)	The use of Pembrolizumab combined with chemotherapy has shown significant effectiveness in treating advanced endometrial cancer. Studies have indicated that the risk of disease progression or death is reduced by 70% with the use of Pembrolizumab.	(41)
Breast cancer	DNA hypomethylation and microRNA interference, based on specific Cytosine-Phosphate-Guanine (CpG) methylation epimutations, affect histone modifications, chromatin remodeling, and DNA methylation	Antibody-drug conjugates (ADCs) exhibit high specificity and targeting precision, demonstrating significant therapeutic efficacy for patients with overexpression of the human epidermal growth factor receptor 2 (HER2) subtype.	(42, 43)
Uterine fibroid	Induces DNA hypermethylation	The use of combination therapy, including the oral gonadotropin-releasing hormone receptor antagonist relugolix, estradiol, and norethindrone acetate, administered once daily, has been effective for women with uterine fibroids and heavy menstrual bleeding, while avoiding side effects associated with estrogen deficiency.	(44, 45)
Ovarian cancer	Affects DNA methylation and dysregulation of lncRNAs	Antibody-drug conjugates (ADCs), such as mirvetuximab soravtansine, have demonstrated significant efficacy in the clinical management of folate receptor alpha (FRα)-positive ovarian cancer patients	(46)

In cancer research, aberrations in epigenetic modifications have been observed across various types of tumors, including anomalies in DNA methylation, histone modifications, and non-coding RNAs (ncRNAs) expression. These abnormal epigenetic changes can result in the overactivation of oncogenes or silencing of tumor suppressor genes, thereby promoting the growth, proliferation, and metastasis of cancer cells. Understanding the roles and mechanisms of these epigenetic modifications in cancer development is crucial for developing personalized and precise treatment strategies that can improve treatment outcomes and patient survival rates.

### DNA methylation

**Figure 1:**
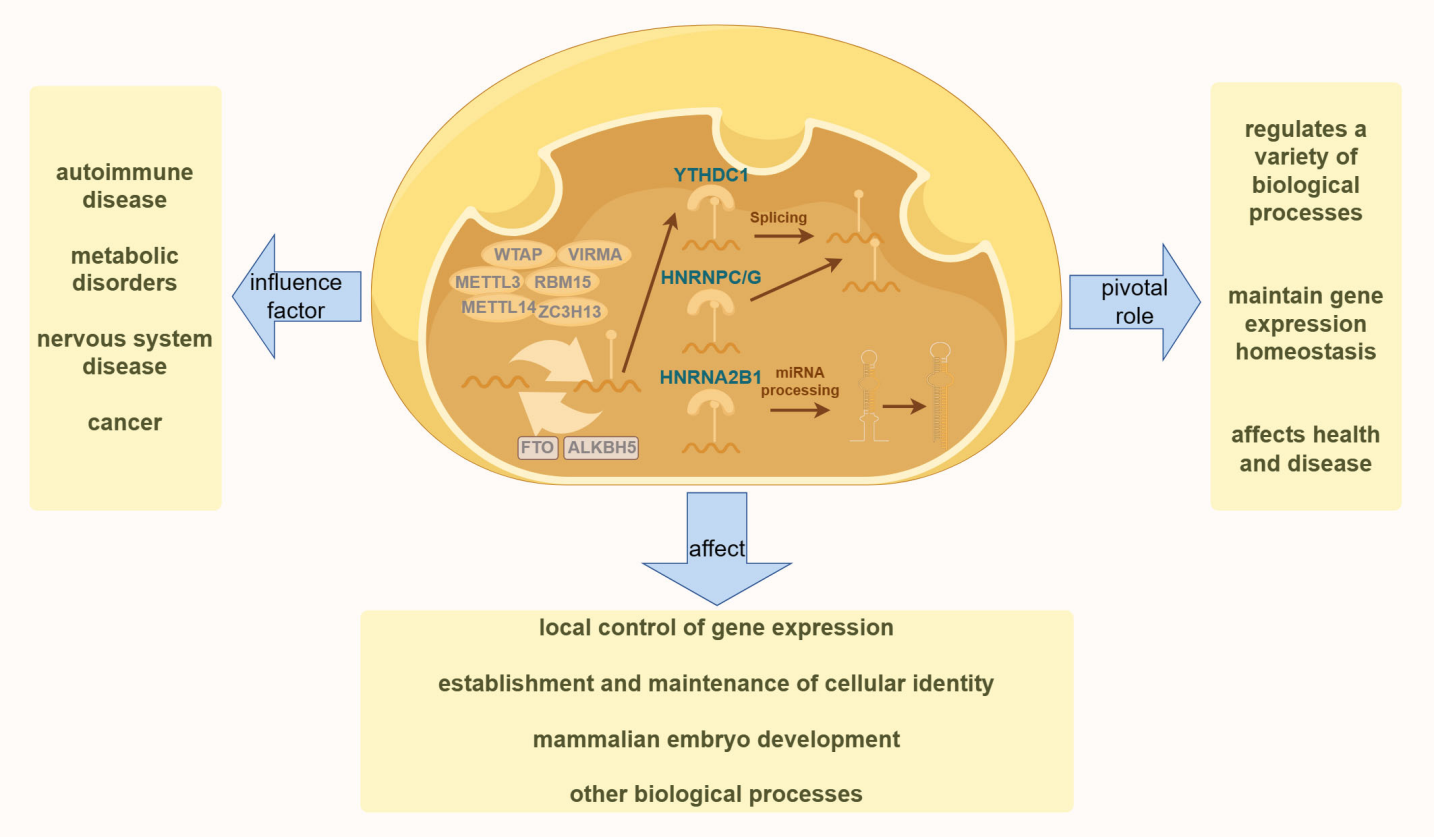
The diagram illustrates the fundamental process of DNA methylation in cells. The left side highlights various human diseases resulting from the dysregulation of DNA methylation. The right side demonstrates the essential role of DNA methylation in various physiological processes in the human body. The bottom section presents the effects of DNA methylation on various aspects of human health.

DNA methylation is a biochemical process in which methyl groups are covalently added to the 5-carbon of the cytosine ring of DNA molecules. This modification occurs in Archaea, bacteria, and eukaryotes. In eukaryotes, DNA methylation is a crucial epigenetic modification that regulates various biological processes, maintains gene expression stability, and affects health and disease (47). As one of the most important epigenetic markers, DNA methylation is reversible and mitotically heritable. It plays a key role in controlling local gene expression (48, 49) establishing and maintaining cellular identity (50, 51) and regulating mammalian embryonic development (52, 53) among other biological processes (54) (55). This includes imprinting, X-chromosome inactivation, and the silencing of repetitive DNA elements (49). As illustrated in **Figure 1**, dysregulation of DNA methylation is associated with a range of human diseases, including autoimmune disorders, metabolic dysfunction, neurological conditions, and cancers (56).

Abnormal patterns of DNA methylation are hallmark features of cancer cells and are believed to be associated with tumorigenesis. High levels of methylation, often observed in transcriptional regulatory elements such as gene promoters and enhancers, particularly those of tumor suppressor genes, suggest that epigenetic mutations may play a critical role in tumor development. Numerous cancer suppressor genes silenced by high DNA methylation have been identified in tumor tissues (57). This methylation can lead to transcriptional silencing of these genes, thereby promoting malignant transformation. Moreover, DNA methylation can occur at various gene loci with different degrees and locations, potentially resulting in distinct regulatory effects on gene expression and biological processes. This differential methylation regulatory mechanism is crucial for normal cellular function and the onset of cancer (58).

It is estimated that 70%–80% of Cytosine-Phosphate-Guanine (CpG) sites in the mammalian genome are methylated (59), except in specific regions known as CpG islands (CGIs). CGIs are CpG-rich sequences that are approximately 1,000 bases long and are predominantly located at gene promoters (60, 61). Approximately 60% of human gene promoters contain CGIs (62). DNA methylation is established by DNA methyltransferases (DNMTs), with DNMT3A and DNMT3B being essential for initiating DNA methylation, whereas DNMT1 maintains methylation during DNA replication (63). The Ten-eleven translocation (TET) family of enzymes, comprising TET1, TET2, and TET3, initiates repair mechanisms by oxidizing 5-methylcytosine (5mC). This oxidation can lead to either replication-dependent dilution or base excision repair, depending on the involvement of thymine DNA glycosylase (TDG) (64). These processes counteract the actions of members of the DNMT family, thereby promoting active DNA demethylation. Genome-wide analyses revealed distinct DNA methylation patterns across different cell types, developmental stages, and responses to various stimuli (65).

Abnormal DNA methylation patterns are associated with various diseases, including cancer (66). In cancer cells, overall DNA methylation levels are often reduced, whereas CGIs are hypermethylated in a cancer-specific manner (67, 68). Histone modifications, chromatin remodeling, and transcription factors play crucial roles in regulating both genome-wide and site-specific DNA methylation (69, 70). This highlights the significant impact of epigenetic changes on cancer development and underscores the potential of targeted epigenetic therapies.

### Histone modifications

In every cell, DNA is packaged in histones to form nucleosomal core particles. These histones have tails with numerous residues that can undergo post-translational modifications (PTMs). Such modifications are crucial, as they influence chromatin architecture and nucleosome dynamics while regulating transcription and affecting essential processes such as DNA repair, replication, stability, and cellular state transitions. Recent studies have highlighted that modifications in the core regions of histones, in addition to the tail regions, play a significant role in directly impacting DNA-based biological processes, including transcriptional regulation and maintenance, genomic stability, and cellular functionality (71).

Histone PTMs are essential for various critical processes in an organism. Aberrant histone modifications are closely linked to the development of diseases, such as cancer. Research has demonstrated that histones can undergo at least 11 types of PTMs on more than 60 different amino acid residues. These include methylation, acetylation, propionylation, butyrylation, formylation, phosphorylation, ubiquitination, SUMOylation, guanidinylation, prolyl isomerization, and Adenosine Diphosphate-ribosylation (ADP-ribosylation). These diverse modification patterns can regulate the structure and function of histones, thereby influencing gene expression, chromatin structure, and overall cellular functionality (72).

Histone H1, the most differentiated and heterogeneous of the histones, undergoes PTMs that are associated with cancer, autoimmune diseases, and viral infections (73). Phosphorylation sites on histone H1 subtypes, particularly H1.1–H1.5 in breast cancer cell lines, are highly conserved among these variants. Studies suggest that phosphorylation primarily occurs in H1.2, H1.3, and H1.5 subtypes, with focal adhesion kinase (FAK) potentially catalyzing this modification. In breast cancer cells, elevated levels of tyrosine phosphorylation in these H1 subtypes compared to normal cells suggest that this modification is crucial for breast cancer progression. Additionally, H1 tyrosine phosphorylation correlated positively with the proliferative state of cells, indicating that H1Y70p (tyrosine phosphorylation at position 70 of H1) may be a key factor in defining the tumor phenotype. These findings highlight the potential role of H1 subtype tyrosine phosphorylation in cell proliferation and tumor development, with FAK emerging as a significant regulator of this process. This underscores the potential targets for future therapies aimed at breast cancer and other diseases, particularly in developing strategies to inhibit or modulate FAK activity (74).

**Figure 2:**
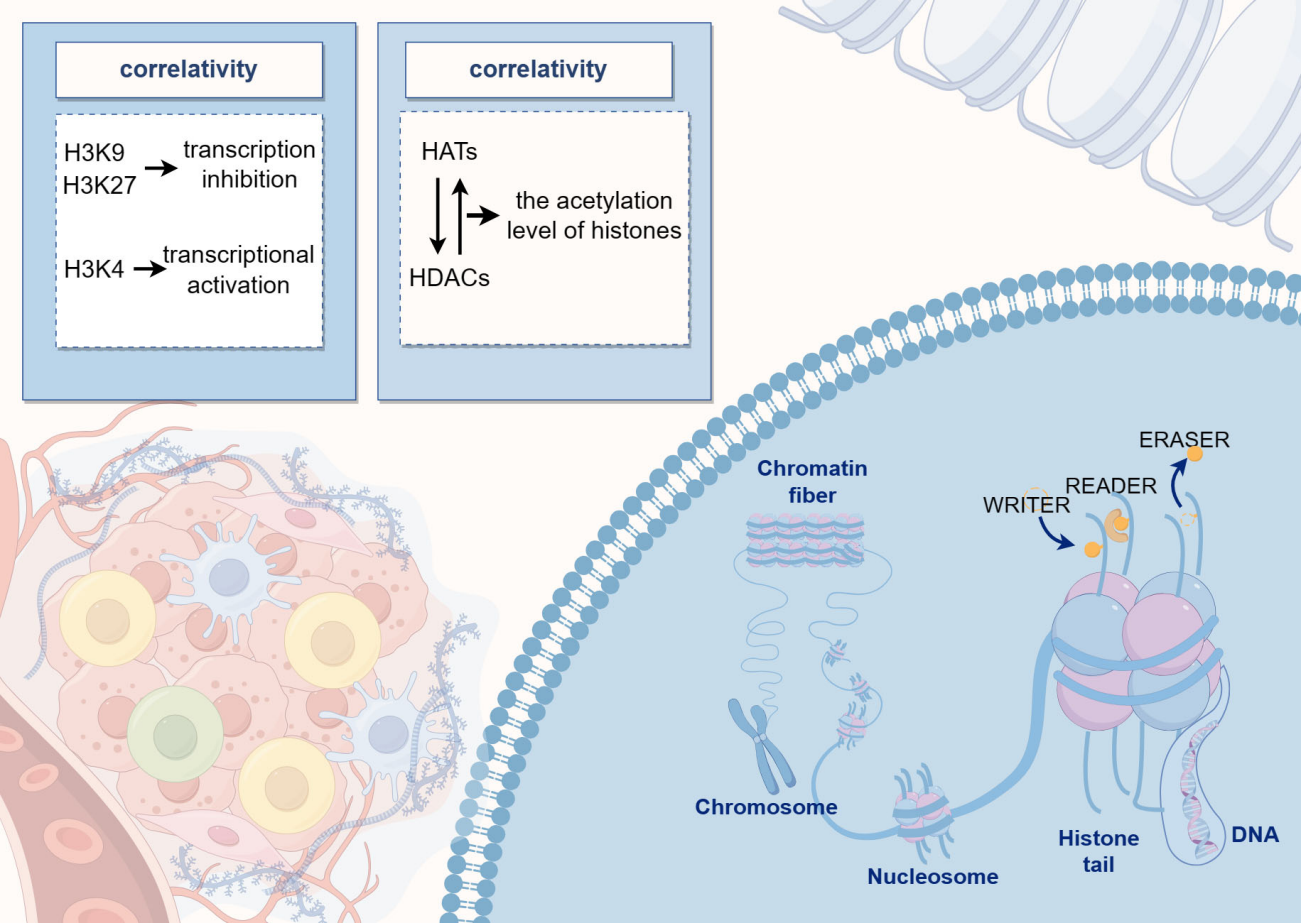
The diagram illustrates the pattern of histone modifications, where DNA binds to histones to form nucleosome core particles, and various residues can undergo post-translational modifications. The center of the image highlights the dynamic balance between histone acetyltransferases and deacetylases, which regulate histone acetylation levels.

Histone acetylation levels are regulated by a dynamic balance between histone acetyltransferases and histone deacetylases (HDACs). Acetylation is generally associated with active gene expression as it leads to the loosening of the chromatin structure, thereby facilitating access to the transcriptional machinery. Conversely, histone methylation can either inhibit or promote transcription depending on the specific amino acid residue that is methylated. For instance, methylation of histone H3 at positions 9 and 27 (H3K9 and H3K27) is typically linked to transcriptional repression, whereas methylation at position 4 (H3K4) is associated with transcriptional activation (**Figure 2**) (75). Methylation is a common modification process that occurs on lysine and arginine residues, particularly at multiple sites on the N-terminal tails of histones. This modification can range from unmethylated to tri-methylated states, with each added methyl group potentially altering the functional characteristics of the genomic regions. Histone methyltransferases are responsible for adding methyl marks to the genome, whereas histone demethylases remove these marks. The balance between these processes influences chromatin structure and gene expression, thereby regulating the activity or silence (76). Both epigenetic and genetic alterations play a critical role in determining cell fate, contributing to cellular homeostasis, or promoting tumor genesis (77).

### Non-coding RNA

In eukaryotic genomes, approximately 90% of genes are transcribed, yet only 1%–2% of these transcriptional products encode proteins. Most transcribed genes produce ncRNAs, which, while transcribed from the genome, do not code for proteins. ncRNAs can be broadly categorized into two types: structural ncRNAs, which are integral to fundamental cellular architecture, and regulatory ncRNAs, which play roles in gene regulation. Based on their length, ncRNAs are classified as small ncRNAs (sncRNAs, 18–200 nt) and long ncRNAs (lncRNAs, > 200 nt).

The biogenesis and function of Small Interfering RNAs, MicroRNAs, and Piwi-interacting RNAs (siRNAs, miRNAs, and piRNAs) in eukaryotes have been extensively studied (78, 79). Both siRNAs and miRNAs are derived from double-stranded RNA precursors, primarily processed by RNase III enzymes, Dicer for siRNA, and both Drosha and Dicer for miRNA (78). In contrast, piRNA, predominantly found in animal germ cells, is produced from single-stranded RNA precursors through a process independent of Dicer and Drosha, involving a set of proteins for primary processing and an amplification mechanism known as the “ping-pong cycle” (80). The primary functions of siRNAs, miRNAs, and piRNAs depend on their base pairing with RNA and/or DNA targets, mediated by Argonaute (AGO) family proteins. These interactions lead to RNA-silencing effects, including post-transcriptional mRNA cleavage, decay, translational repression, and transcriptional silencing. siRNAs and miRNAs are associated with the AGO subfamily, whereas piRNAs are linked to the P-element-induced Wimpy Testis (PIWI) subfamily. Notably, AGO-dependent RNA silencing is generally considered to occur exclusively in eukaryotes (81).

lncRNAs are emerging as significant regulatory factors involved in gene expression and various physiological and pathological processes (82). Increasing evidence has highlighted their critical role in cancer formation and progression. lncRNAs can act as oncogenes or tumor suppressor genes, providing complex and precise regulation of cancer cell behavior, including proliferation, differentiation, invasion, and metastasis. Additionally, they play a role in modulating metabolic reprogramming in cancer cells (83) (84). lncRNAs are also crucial in regulating the transcription and translation of metabolism-related genes. They may function as molecular “decoys,” “scaffolds,” or competitive endogenous RNAs, influencing cancer metabolic reprogramming and contributing to the complexity of cancer metabolism (85).

tRNA-derived small RNAs (tsRNAs) originate from tRNA precursors in the nucleus and are frequently dysregulated in various cancers, particularly gynecological malignancies. tsRNAs can bind to both AGO proteins (such as miRNAs) and PIWI proteins (such as piRNAs), thereby playing significant regulatory roles in gene expression. They participate in both pre-transcriptional regulation (such as piRNAs) and post-transcriptional regulation (such as miRNAs). Similar to piRNAs, tsRNAs are single-stranded molecules that can interact with DNA and histone methylation mechanisms, indicating their role in the pre-transcriptional regulation of gene expression. Like miRNAs, ts-53 (formerly known as miR-3676) can interact with the 3’ untranslated region (3’ UTR) of TCL1, supporting its role in the post-transcriptional regulation of gene expression. Dysregulated tsRNAs have significant impacts on various malignancies, including gynecological cancers, and the biological functions of tRFs are Ago-dependent. In ovarian cancer, tRF5-Glu regulates the levels of Breast Cancer Anti-Estrogen Resistance 3 mRNA by directly binding to the 3′ UTR (86). In patients with colorectal, breast, and ovarian cancer and their corresponding cell lines, the expression levels of ts-101 and ts-46 are associated with chromatin structure, cell survival, proliferation, clonal growth, and apoptosis. Besides, the expression of tRFs is linked to oncogene activation and ovarian cancer progression (87). Reanalysis of existing RNA sequencing data from 180 serum samples, including 15 healthy controls, 46 benign, and 22 borderline tumors, and 97 patients with ovarian cancer, revealed that tsRNAs constituted a significant proportion of total small RNAs (ranging from 2.5% to 29.4%) and were not random degradation products in serum but were enriched for several specific types of related tRNAs (for instance, Glycine-Transfer RNA), which can predict abnormal cell proliferation with high accuracy (88). Another group using serum samples from ovarian cancer patients, healthy donors, and ovarian cancer cell lines exhibited differential expression of tRFs; the results indicated that tRF-03357 promoted proliferation, migration, and invasion of Ovary Adenocarcinoma 3 (SK-OV-3) cells and downregulated Homeobox Containing 1 (HMBOX1) (89). In cervical cancer, preliminary studies using biopsy samples exhibited significantly elevated expression of 5S Ribosomal RNA (5S rRNA), Transfer RNA (tRNA) arginine, and tRNA Sec in samples containing Human Papillomavirus Type 16 (HPV16) compared to HPV-negative biopsies (90). The role of tRNA/tiRNAs in endometrial cancer remains unexplored. However, these findings suggest that tsRNAs play important roles in gene expression regulation and may be key regulators of the onset and progression of cancer. Further research will help elucidate the specific functions and mechanisms of tsRNAs in cancer and provide a theoretical basis for developing tsRNA-based therapeutic strategies. Research on tsRNAs can deepen our understanding of the complexities of gene expression regulation and offer new targets and strategies for future cancer treatment (90, 91).

### Epigenetic regulation of gene expression mechanisms

**Figure 3:**
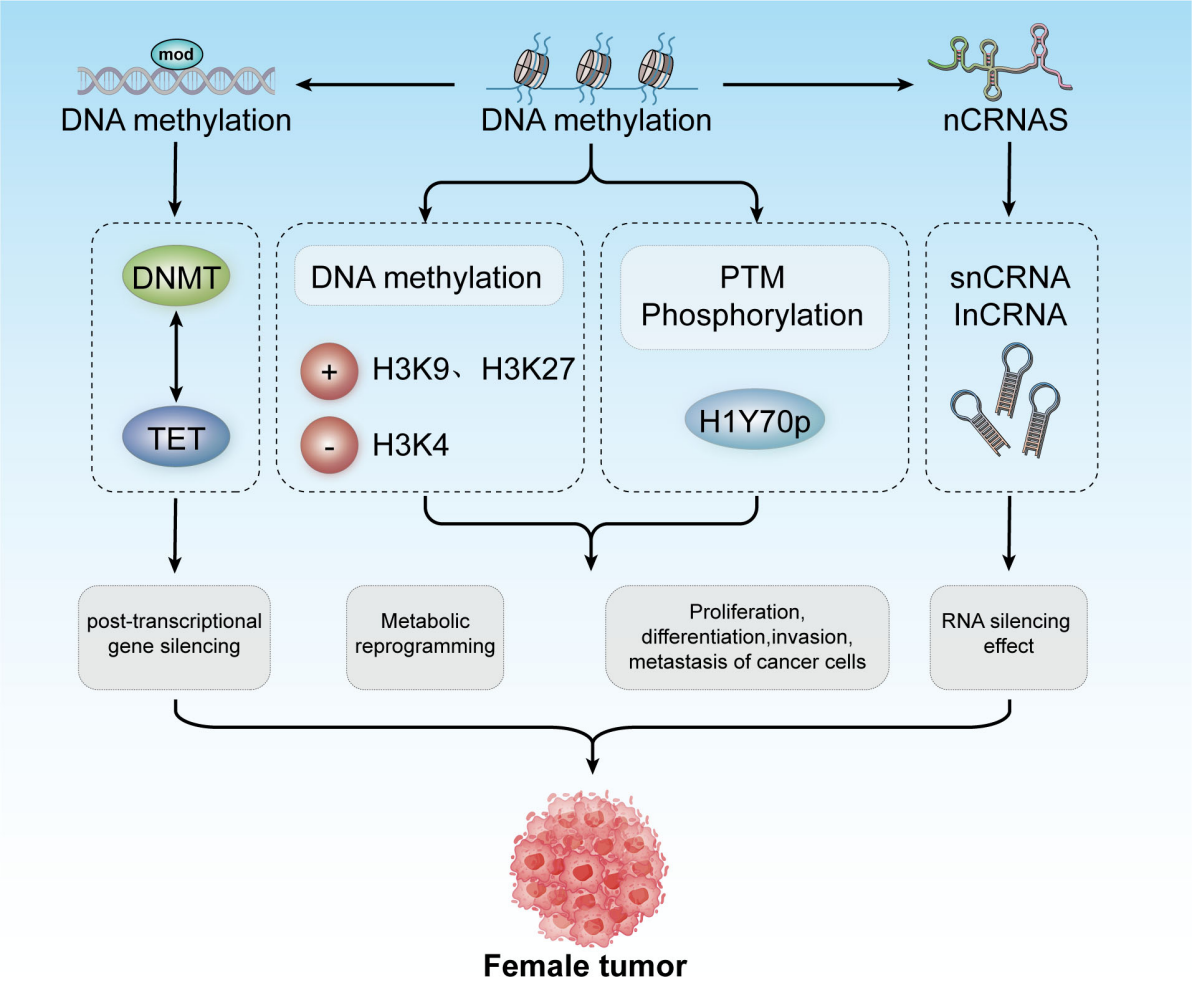
Epigenetic modifications include DNA methylation, histone modification, and non-coding RNA mechanisms. These modifications contribute to post-transcriptional gene silencing, metabolic reprogramming, and regulation of cancer cell proliferation, differentiation, invasion, and metastasis. Collectively, these processes play a role in developing tumors in females.

In 1942, Conrad Waddington coined the term “epigenetics” to describe heritable changes in phenotypes that occur without alterations in the genotype (92, 93). Today, epigenetics broadly refers to ‘heritable phenotypes arising from changes in chromosomes without altering the DNA sequence’ (94). Epigenetic regulation is crucial for development, cell fate determination, proliferation, genomic integrity, and fundamental transcriptional control. This regulation occurs at multiple levels, including DNA methylation, histone modifications, nucleosome remodeling, and regulation of the three-dimensional chromatin structure (**Figure 3**) (95). The connection between cancer genetics and epigenetics is evident in the abnormal metabolic and biochemical pathways observed in cancer, along with mutations in genes that function as epigenetic regulators. Reversing these epigenetic changes has depicted significant efficacy in treating the early stages of various types of lymphomas and leukemia, with similar therapeutic outcomes observed in solid tumors (96). For instance, activation of Wingless-type (WNT) signaling is linked to the onset and progression of various cancers, including breast cancer. The epigenetic silencing of WNT antagonist genes (such as SFRP and DKK) contributes to the initiation of breast tumors (97). Mechanistically, silencing of these genes through DNA methylation is a principal cause of continuous WNT signal transduction in breast cancer and is associated with poor prognosis (98). These changes result in constitutive activation of β-catenin, leading to increased stem cell renewal and proliferation, which is linked to disease recurrence. In a study of 96 breast cancer samples, promoter methylation of the Dickkopf (DKK) family member DKK3 was found to be significantly enriched in tumors from patients with advanced disease, lymph node metastasis, and positive Estrogen Receptor alpha (ERα) status (42 of 47 samples were ER^+^) (99).

The repressive state of chromatin can be maintained throughout the cell cycle through specific histone modifications, DNA methylation, regulatory proteins, and non-coding RNAs (100). In multiple human cancers, the loss of function of active chromatin remodelers results in a more compact chromatin state. Moreover, in many tumor types, specific CpG island hypermethylation suppresses the expression of tumor suppressor genes (such as p16) and DNA mismatch repair genes (such as MLH1 and MSH2), thereby promoting cancer progression (101). In Isocitrate Dehydrogenase-mutant (IDH-mutant) gliomas, extensive DNA hypermethylation reduces the binding capacity of the transcriptional repressor, CCCTC-binding Factor (CTCF), leading to impaired insulator function. These functions are crucial for the regulation of gene expression (102). Various cues can trigger abnormal changes in chromatin state, making it either open or closed. These changes may activate oncogenes or disabled tumor suppressor genes, endowing cells with the six essential hallmarks of cancer (103).

## Epigenetic alterations in cancers in females

### Epigenetic markers in various cancers in females

**Figure 4:**
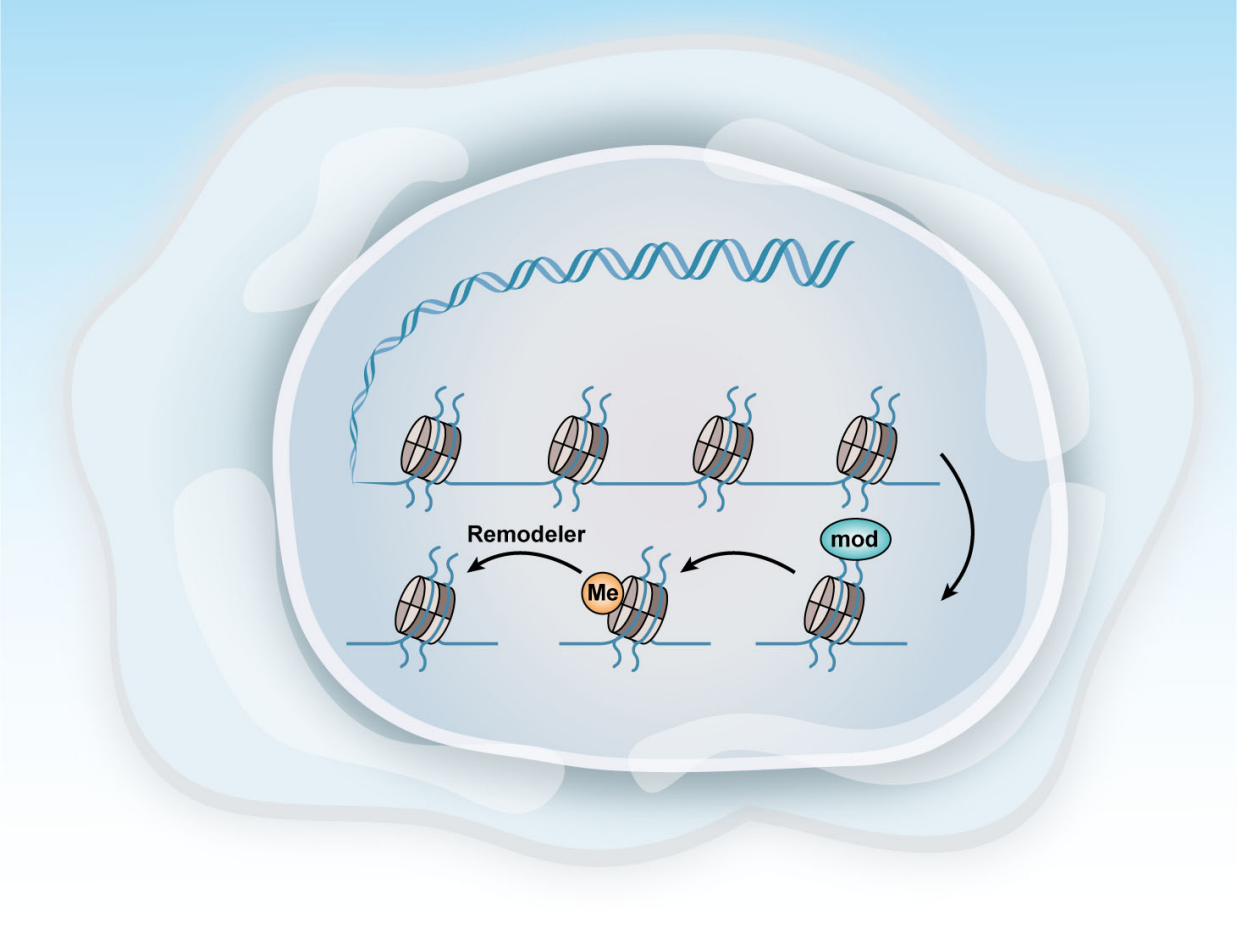
Methods of epigenetic regulation. Epigenetics can be regulated at various levels. This figure illustrates three key epigenetic examples: histone modification, DNA methylation, and chromatin remodeling.

Ovarian cancer, like many other cancers, is characterized by alterations in various epigenetic regulators, including Enhancer Of Zeste Homolog 2,SNF-related, Matrix-associated, Actin-dependent Regulator of Chromatin2/4, and AT-rich Interactive Domain-containing Protein 1A (EZH2, SMARCA2/4, and ARID1A). Dysregulation of these factors frequently disrupts transcriptional control mechanisms, leading to abnormal cell fate decisions and disturbances in the pathways related to cell senescence, death, and proliferation. These epigenetic modifications, often driven by mitotic processes, are considered promising therapeutic targets because of their reversible nature (**Figure 4**) (104).

A meta-analysis involving 43 studies and 16,336 women found that DNA methylation testing exhibited higher specificity than HPV16/HPV18 genotyping or cytology tests used to identify ≥ Atypical Squamous Cells of Undetermined Significance (ASCUS) level atypical squamous cells. This makes DNA methylation testing a more effective tool for further triaging after the initial screening (105). Among women with high-risk HPV genotypes detected in cervical samples, DNA methylation testing for ≥ Cervical Intraepithelial Neoplasia Grade 2 (CIN2) demonstrated a relative sensitivity of 1.22 (95% confidence interval [CI]: 1.05–1.42) compared to HPV16/HPV18 genotyping and 0.81 (95% CI: 0.63–1.04) compared to ≥ ASCUS cytology. The relative specificities were 1.03 (95% CI: 0.94–1.13) and 1.25 (95% CI: 0.99–1.59), respectively.

Pituitary Homeobox 2 (PITX2) methylation predicts outcomes of adjuvant anthracycline chemotherapy in high-risk patients with lymph node-positive, estrogen receptor (ER) positive, and HER2-negative breast cancer (106, 107). The Therascreen PITX2 RGQ PCR test (QIAamp Cador Pathogen Mini Kit), a Capillary Electrophoresis-marked assay, is highly reliable and robust for determining the PITX2 promoter methylation status. This test is effective in predicting outcomes in high-risk patients with breast cancer undergoing anthracycline-based chemotherapy (hazard ratio 2.48; p < 0.001) (108).

### Relationship between epigenetics and cancer onset and progression

Epigenetic modifications are defined as inheritable changes in gene activity that occur without alterations to the underlying DNA sequence (3). Fine-tuning gene expression programs through epigenetic factors is the principal molecular mechanism controlling key biological processes, such as cell differentiation and embryogenesis. Compelling evidence suggests that epigenetic reprogramming drives dynamic transcriptional heterogeneity observed in cancer (4). Among the various types of epigenetic modifications, DNA methylation is the most extensively studied in humans. Since its discovery in primary human tumors four years ago, comprehensive research has robustly demonstrated that changes in DNA methylation patterns are instrumental in orchestrating cancer progression and metastasis (5).

Distortions in epigenetic mechanisms can affect a wide range of physiological processes and often lead to pathological conditions (109). Various disease categories, including neuropathology (110) and oncology (111), have been associated with misregulated epigenetic control. In the context of cancer, epigenetic alterations are classified into three distinct yet interconnected categories: epigenetic regulators, modifiers, and mediators (112). Epigenetic modifiers are enzymes or protein complexes that directly add or remove chemical modifications to DNA or histones, making them critical targets for cancer development. Epigenetic mediators are responsible for transmitting epigenetic information or signals, usually working in conjunction with modifiers or during the subsequent phases of their effects. Epigenetic regulators are positioned upstream of modifiers, directing their activity and localization and disrupting specific epigenetic patterns linked to cellular differentiation (113). Abnormalities in these mechanisms, which act as connectors between environmental factors and the epigenome, increase susceptibility to cancer and accelerate its progression. The increasing focus on epigenetic alterations in cancer research, particularly their role in driving cancer hallmarks, has significantly influenced both research and therapeutic approaches (4).

## Transcriptomics and epigenetics

### Applications of transcriptomics in epigenetics

Whole transcriptome analysis is essential for understanding genomic structure and function, identifying the genetic networks underlying cellular, physiological, biochemical, and biological systems, and establishing molecular biomarkers for diseases, pathogens, and environmental challenges (114). This approach aims to capture and quantify gene expression heterogeneity across various levels, from individual cells to tissues, organs, and the entire organism. It represents a critical initial step in characterizing and annotating the functions of genes or genomes revealed through DNA sequencing (115).

RNA methylation—a widespread phenomenon in both eukaryotes and prokaryotes—is a significant focus in epigenetics. To investigate cytosine methylation in RNA, several RNA sequencing-based techniques have been developed to detect methylation sites with single-nucleotide precision, with or without chemical treatment. One such method, bisulfite sequencing of RNA, is analogous to bisulfite sequencing used for DNA. This technique involves treating RNA with bisulfite to convert methylated cytosine into uracil. Schaefer and his team successfully utilized this method in combination with high-throughput sequencing to reveal RNA methylation patterns (116). For instance, Khoddami and colleagues employed two mammal-specific cytosine RNA methyltransferases (m5C-RMTs) and the cytosine analog 5-azacytidine (117, 118). They developed a method known as Aza-immunoprecipitation (Aza-IP), which stabilizes m5C-RMT and RNA binding in cell culture. These complexes were then extracted through immunoprecipitation and analyzed using high-throughput sequencing to study RNA methylation patterns. Similarly, the RNA methyltransferase Nsun2 has been utilized to develop a methylation individual-nucleotide-resolution crosslinking and immunoprecipitation method that detects cytosine methylation in RNA species (119).

### Advances in sequencing technologies in cancer research

#### RNA sequencing

The transcription and stability of RNA are tightly regulated by both physiological and pathological stimuli (120). Aberrant RNA expression is often associated with the onset, development, progression, and metastasis of human cancer. Beyond mutations in tumor suppressor genes and oncogenes, gene expression can be either over-activated or epigenetically silenced, potentially leading to uncontrolled growth and proliferation of tumor cells. Abnormal activation of cellular growth signaling pathways or transcription factors may result in high-level expression of genes linked to tumor development and progression. Distinct gene expression profiles can reflect various cancer subtypes, stages of cancer development, or the tumor microenvironment (121–123). Therefore, RNA sequencing is a powerful tool for elucidating the molecular mechanisms underlying cancer development and for developing new strategies for cancer prevention and treatment (124). This technique has been extensively applied in cancer research and treatment. This includes identifying and characterizing biomarkers for cancer heterogeneity and evolution, studying mechanisms of resistance, exploring the cancer immune microenvironment and immunotherapies, and identifying new cancer antigens (125). RNA sequencing is widely used for cancer classification, biomarker and gene fusion discovery, disease diagnostics, and therapy optimization. Translational oncology research focuses primarily on two areas. The most widely used is for the identification of biomarkers for cancer diagnosis, prognosis, and prediction. Various RNA seq-based features have been developed and validated across numerous primary cancer types (126–130). Differential gene expression analysis is one of the most common applications of RNA sequencing (131), which enables the comparison of samples from different backgrounds (such as species, tissues, and time points) to identify differentially expressed genes, thereby unveiling their functions and potential molecular mechanisms. This analysis also aids in the discovery of potential cancer biomarkers (132, 133). Gene fusions, which are closely related to tumorigenesis, have been demonstrated to be ideal cancer biomarkers and therapeutic targets (134).

**Figure 5:**
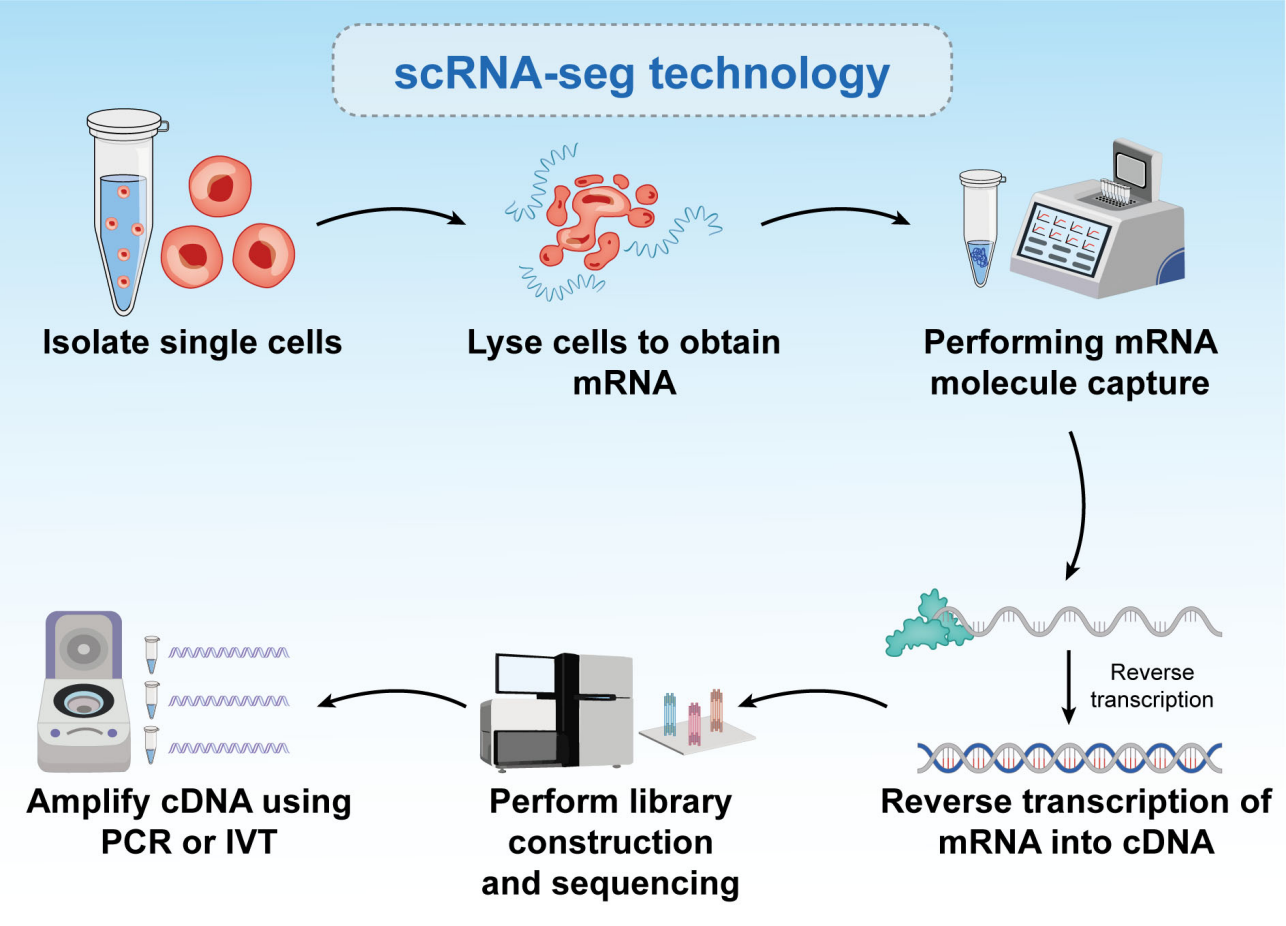
Six-step process of single-cell RNA sequencing. The figure is a brief description of single-cell sequencing, and the text is a brief description of the steps. Single-cell sequencing can reflect the differences between different cells.

Single-cell RNA sequencing (scRNA-seq) is a powerful technique for the characterization of individual cells. Unlike traditional bulk RNA sequencing, which measures average gene expression across a sample and identifies differences between sample conditions, scRNA-seq measures gene expression levels at the single-cell level. This allows for the identification of differences between cells within one or more samples, revealing potential heterogeneity and functional differences within cell populations (135). scRNA-seq technology has significantly advanced cancer research by enabling the detection of subpopulations of cancer stem cells, metabolic shifts in cancer-draining lymph nodes, and therapy-induced adaptations in cancer cells (136). Combining scRNA-seq with parallel Clustered Regularly Interspaced Short Palindromic Repeats (CRISPR) screens allows for the simultaneous analysis of genomic perturbations and transcriptional activity at the single-cell level. This approach reveals heterogeneous cell types and key factors involved in complex regulatory mechanisms (**Figure 5**) (137).

Advanced protocols such as Switching mechanism at 5’ end of the RNA transcript (Smart-seq) (138) and Smart-seq2 (139) facilitate work with very small amounts of starting mRNA, which can be amplified from single cells. These protocols enable the creation of single-cell libraries that uncover new and previously uncharacterized cell types within tissues. They also explored interesting phenomena in molecular biology, such as the stochasticity of gene expression among similar cell types within a population. By comparing single-cell data with cell populations, researchers can identify multiple subpopulations with different gene expression profiles. These differences may be attributed to natural factors, such as varying stages of the cell cycle, or reflecting rare cell types, such as cancer stem cells. Recent advancements in single-cell preparation methods, including platforms such as Fluidigm C1, have enhanced the analysis of larger numbers of individual cells, ranging from a few to 50–90 cells at a time and up to 800 cells. This progress has greatly improved our understanding of cell populations. Other methods, such as Droplet sequencing (140), can simultaneously analyze over 10,000 cells. Increasing the number of single-cell libraries per experiment directly aids in identifying smaller subpopulations within cell populations.

#### Chromatin immunoprecipitation sequencing

ChIP-seq is a core method in epigenomic research. This technique uses antibodies specific to certain DNA-binding proteins or histone modifications to identify enriched sites across the genome (141). By analyzing histone modifications using ChIP-seq, researchers can gain in-depth insights into epigenetic features and their roles in various biological functions. Whole-genome analysis of histone modifications, such as enhancer analysis and genome-wide chromatin state annotation, has systematically investigated how epigenetic landscapes affect cellular development, lineage specification, and disease-related processes (142). Recent advances in next-generation sequencing technologies and computational analyses have significantly enhanced our understanding of how epigenetic landscapes contribute to cell identity (143), development (144), lineage specification (145), cancer (146), and other diseases (147).

Phenotypic changes crucial for normal development and disease are temporally and spatially regulated by chromatin-coordinated gene expression (148). DNA-protein interactions play a pivotal role in cellular phenotypes and have been extensively studied using various biochemical and genomic methods. Traditional techniques, such as electrophoretic mobility shift assay (EMSA) and Deoxyribonuclease I (DNase I) footprinting, have been valuable; however, their *in vitro* application limits their ability to reflect the cellular context. This limitation has driven the development of new methods for analyzing DNA-protein interactions in living cells. ChIP has emerged as a popular technique for identifying genomic regions associated with specific proteins in their native chromatin environment. ChIP enables the capture of protein-DNA binding sites, consequently detecting DNA-protein interactions in living cells and overcoming some drawbacks associated with EMSA and DNase I footprinting. Using ChIP, researchers can determine where various transcription factors, histones, and other proteins bind within the genome, offering significant insights into genomic regulation and epigenetic mechanisms. ChIP identifies specific genomic sites with which proteins interact, revealing the molecular mechanisms that regulate gene expression. This technique provides crucial information for understanding the formation and regulation of cellular phenotypes and has substantial potential for advancing disease research and drug development (149–153).

Gilmour and Lis developed the initial ChIP technique while studying the association of RNA polymerase II with the transcription and localization of genes in *Escherichia coli* and fruit flies (154–156). They used ultraviolet light to covalently crosslink proteins to adjacent DNA in intact living cells. Solomon and Varshavsky later replaced ultraviolet crosslinking with formaldehyde crosslinking (157).

Compared to bulk ChIP-seq, which cannot capture single-cell chromatin features, single-cell ChIP-seq (scChIP-seq) offers a powerful approach to studying the genetic diversity within heterogeneous cell populations and understanding the evolution of tumor populations. Droplet-based single-cell ChIP-seq integrates microfluidic technology with single-cell DNA to provide a relatively low coverage map for each cell (158). scChIP-seq enables clustering of cell populations based on chromatin landscape diversity and identifies chromatin features specific to each group, such as the absence of the Histone 3 K27 Tri-methylation marker in some cells, which may be linked to chemotherapy resistance (159). By combining ChIP-seq with other techniques, such as ATAC-seq and DNA mutation profiling in the same cells, researchers can uncover new subclones of cancer cells, paving the way for personalized clinical trials. Consequently, understanding chromatin at the single-cell level has significantly advanced biomedical research in cancer therapy (160). Despite these advantages, ChIP-seq faces several challenges. PCR amplification can introduce bias, and the length of amplification is limited. Furthermore, fragmentation and sequencing processes may be affected by Gas Chromatography content bias. Significant cell loss during immunoprecipitation often requires many cells (10^5^–10^7^). The formaldehyde crosslinking step can also obscure specific binding sites, potentially affecting the accuracy of the experimental results (161).

Initially, the detection of specific protein-DNA interactions relied on methods such as Southern blot hybridization or PCR-based amplification, which evaluate interactions by assuming that specific target sequences interact with purified chromatin components. However, the advent of DNA microarray technology has marked a significant advancement, enabling simultaneous detection of multiple sequences. This capability has greatly expanded the genomic scale that could be studied in a single experiment, allowing researchers to detect thousands of genomic sites at once and accelerate the understanding of specific protein-DNA interactions. Consequently, DNA microarrays provide a more comprehensive view of genome organization and regulation (153) (162). The integration of ChIP with next-generation sequencing has revolutionized this field by enabling genome-wide studies in humans (163) (145) (164). In ChIP-seq, a specific antibody is used to identify all interactions of a target factor across the genome, allowing for easy adjustment of experiments to compare different conditions and to understand transcription dynamics. As technology matures, attention has shifted toward developing bioinformatics strategies to analyze the vast genomic-scale data generated by ChIP-seq experiments (165). New experimental techniques, such as Cleavage Under Targets and Release Using Nuclease (CUT&RUN) (166) and Cleavage Under Targets and Tagmentation (CUT&Tag) (167), have addressed the biases and background issues inherent to standard ChIP-seq experiments. These methods provide a single-base pair resolution and require smaller sample inputs, consequently generating more reliable data. The development and application of ChIP-seq and related technologies in transcription regulation research are detailed in previous studies (168–172).

**Figure 6:**
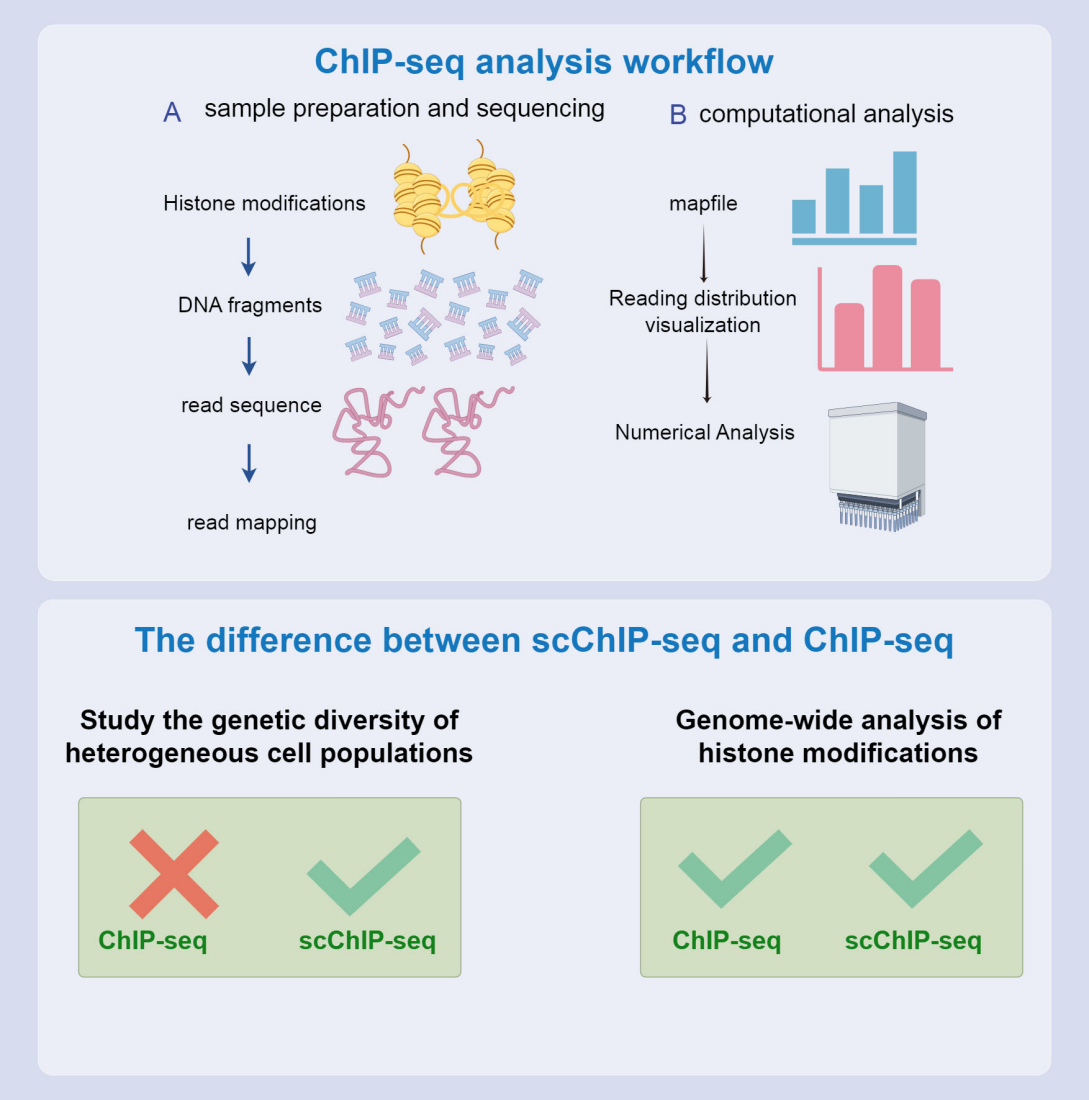
CHIP-seq process and differences from scCHIP-seq. The top and bottom half of the flow chart compare the two. Both can be used to study epigenetic characteristics; however, scCHIP-seq is more useful for studying the genetic diversity of heterogeneous cell populations.

Although ChIP-seq is an effective tool for revealing genomic structures and functional elements, it faces several experimental design limitations that pose challenges. One significant limitation is reliance on high-quality antibodies. This dependence restricts the range of factors that can be studied and impedes the discovery of novel genomic regulators. Although ChIP-seq can identify genome-wide binding sites for specific factors, it provides limited information regarding the background context of these sites and fails to identify auxiliary factors that might bind to specific genomic locations. Current methods also fail to determine site-specific proteomes through affinity purification and mass spectrometry. Although ChIP-seq excels in mapping the genome-wide binding of specific factors, constructing site-specific proteomes remains challenging. To address these limitations and better understand the regulatory environment of specific genomic regions, continuous ChIP-seq experiments are necessary, which require making specific assumptions about potential regulatory factors. As a complementary approach, some laboratories have developed reverse ChIP strategies. These strategies aim to assess regulatory factors at arbitrarily selected genomic sites of interest in an unbiased manner, thereby expanding our understanding of genomic regulation (**Figure 6**, 173).

### Epigenetics in treating cancers in females

#### Current epigenetic-based therapeutic approaches

Recently, the disruption of epigenetic mechanisms has unveiled significant opportunities in the treatment of nearly all human cancers (174). This revelation has unlocked tremendous potential in oncology, as the reversible nature of chromatin states makes epigenetic mechanisms promising targets for cancer therapy. Epigenetics encompasses reversible modifications to DNA and histones that affect gene expression without altering the underlying DNA sequence (175). Disruptions in these epigenetic mechanisms can lead to the aberrant activation or suppression of crucial cancer-related genes, thereby contributing to tumor initiation.

Ovarian cancer frequently remains asymptomatic until it reaches advanced stages, leading to a dismal five-year survival rate of only 30%, which makes it one of the deadliest cancers. Common mutations in ovarian cancer are found in well-characterized tumor suppressor genes such as *p53* and *BRCA1/2*. Recent research has highlighted that, similar to many other cancers, ovarian cancer is marked by alterations in a range of epigenetic regulators, including *EZH2*, *SMARCA2/4*, and *ARID1A*.

The field of CRISPR screening is evolving rapidly, and advancements in techniques such as knockout, activation, interference sequencing, and domain tiling screens have significantly improved our ability to identify specific vulnerabilities in cancer cells (176). Databases such as The Cancer Dependency Map are instrumental in uncovering synthetic lethal targets and providing new and potentially druggable options for treatment (177). The widespread application of these screening techniques in ovarian cancer models, including 2-dimensional cell lines and organoid systems, promises to enhance the identification of drug targets tailored to individual patients and mutation contexts. Over the next decade, these technologies are anticipated to play a crucial role in the advancement of ovarian cancer treatment.

**Figure 7:**
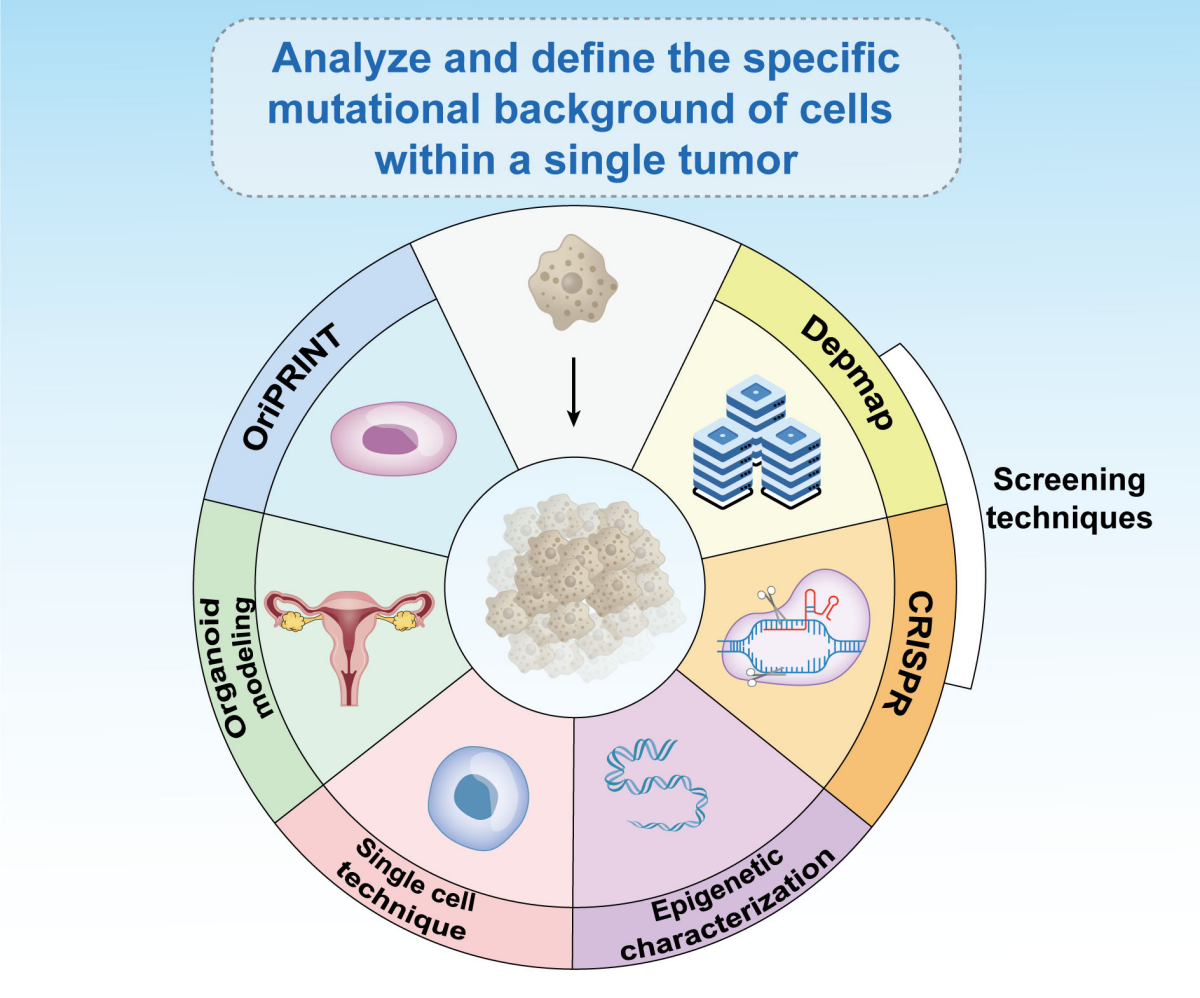
Existing epigenetic-based therapies have broad prospects for development. The analysis and definition of the specific mutational background of cells within a single tumor is critical to the treatment of cancer in women and in general. Emerging fields such as CRISPR screening, databases such as Depmap, OriPRINT technology, organoid modeling, single-cell technology, and epigenetic characterization can help treat tumors.

Recent research has highlighted significant individual variations in mutations and epigenetic alterations, underscoring the need for personalized treatment strategies. What proves effective for one patient may not work for another and could be potentially harmful. An essential step in treating cancers in females and cancer more broadly is to thoroughly analyze and define the specific mutational and epigenetic landscape of cells within each individual tumor (104). Techniques such as OriPRINT can be instrumental in characterizing the cellular origins of different cancer subtypes (178), aiding in the precise molecular characterization and identification of subtype-specific features. Emerging fields, such as organoid modeling, single-cell technologies, and epigenetic characterization, can guide effective treatment approaches (**Figure 7**).

#### Clinical trials and case studies

*In vitro* studies have highlighted the critical role of tumor suppressor genes and DNA repair enzymes in cancer regulation (179). Recent advances have underscored the potential of combining epigenetic drugs in both *in vitro* experiments (180) and clinical trials involving chemotherapy (181). Epigenetic therapies influence various cellular processes, including differentiation, cell cycle arrest, cell death, and energy metabolism, and affect a broad array of genes and proteins (182). These effects are pivotal in cancer progression and contribute to our understanding of the cancer markers related to progression, survival, and regulation (183). Epigenetic-based diagnostic and prognostic tools are integral in precision oncology. Numerous DNA methylation-based diagnostic screenings are either in clinical trials or already in use (184). Research in precision oncology continues to enhance our understanding of epigenetic mechanisms, leading to the development of drugs targeting specific epigenetic regulators. To date, nine epigenetic drugs, including inhibitors targeting EZH2, IDH, and HDACs, have received approval from the Food and Drug Administration. Several other epigenetic drugs are currently undergoing clinical trials.

**Figure 8:**
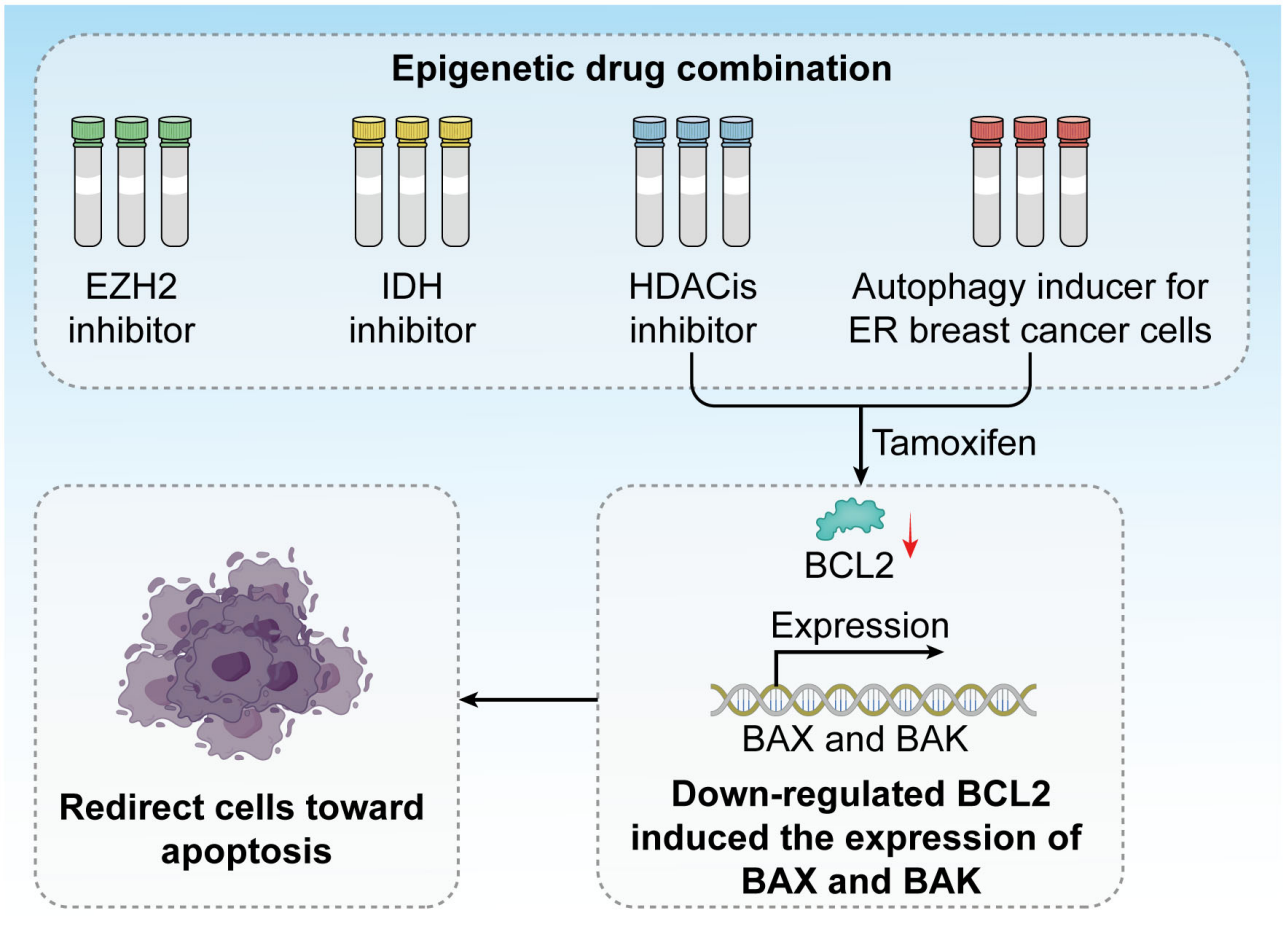
Epigenetic drug combinations have great potential in clinical trials. Inhibitors against EZH2, IDH, and HDACis have been developed, and tamoxifen treatment induces autophagy in ER breast cancer cells. The combination of HDAC inhibitors and tamoxifen redirects these cells toward apoptosis by downregulating BCL2 and inducing the expression of the pro-apoptotic proteins BAX and BAK.

Epigenetic factors play a crucial role in regulating cell death mechanisms, particularly in response to endocrine therapies, such as tamoxifen. Tamoxifen treatment has been demonstrated to induce autophagy in ER-positive breast cancer cells, aiding in their suppression of these cancer cells. By combining HDAC inhibitors with tamoxifen, it is possible to redirect these cells toward apoptosis, primarily through the downregulation of BCL2 and the induction of pro-apoptotic proteins Bcl-2-associated X Protein and BCL2 antagonist/killer (BAX and BAK) (185). This approach has garnered support from several clinical trials exploring the applications of HDAC inhibitors in combination with exemestane and tamoxifen (**Figure 8**).

#### Epigenetic markers in clinical samples

Recent studies have revealed two novel cell death pathways: one called “cuproptosis” and the other referred to as “disulfide apoptosis.” The “cuproptosis” pathway is dependent on copper and involves 12 genes: 7 pro-cuproptosis genes (FDX1, LIAS, LIPT1, DLD, DLAT, PDHA1, and PDHB), 3 anti-cuproptosis genes (MTF1, GLS, and CDKN2A), and 2 key copper transport proteins (ATP7B and SLC31A1). Researchers conducted a comprehensive multi-omics characterization analysis of these 12 genes across over 9,000 samples from 33 cancer types. This study elucidated the characteristics of the cuproptosis genomic landscape in cancer, including single nucleotide variations, copy number variations, methylation, mRNA expression, and miRNA regulation, indicating that genomic alterations and the ectopic expression of miRNA-mRNA networks are associated with the activation of cancer-related pathways (186, 187).

The “disulfide apoptosis” pathway is reliant on disulfide proteins, providing new insights into cell death mechanisms and potentially impacting targeted therapy strategies. This research evaluated the genomic and clinical associations of genes related to disulfide apoptosis (such as SLC7A11, INF2, CD2AP, etc.) in various cancers. The results showed that FLNA and FLNB are the most commonly mutated genes, with the highest mutation rates found in uterine corpus endometrial carcinoma (UCEC) and skin melanoma (SKCM), while mutations in ACTN4 were associated with poorer survival rates in cervical cancer (CESC) and esophageal cancer (ESCA) (188).

In another study, Liu et al. explored the role of voltage-gated sodium channels (VGSCs) in cancer. Although VGSCs primarily trigger action potentials in excitable cells, some VGSC genes are abnormally expressed in cancers derived from “non-excitable” tissues, making them potential therapeutic targets (189).

## Conclusion and future perspectives

### Summary of key points

Epigenetics play a crucial role in the treatment of cancers in females by regulating gene expression, which affects cellular differentiation and proliferation and consequently influences tumor development. Epigenetic modifications consist of three main components: DNA methylation, histone modifications, and non-coding RNA, all of which are primary influencers in tumor genesis. Among the most extensively studied, DNA methylation involves the addition of a methyl group to the 5’-carbon of cytosine residues within CpG dinucleotides. CpG dinucleotides are unevenly distributed across the mammalian genome and are predominantly clustered in CGIs, often found within gene promoters (190). During DNA replication, the TET family of enzymes triggers a series of repair mechanisms by oxidizing 5mC, which may involve replication-dependent dilution or base excision repair by TDG to counteract the actions of DNMTs, consequently promoting active DNA demethylation (58). Increased demethylation activity increases the probability of tumorigenesis. From a histone perspective, modifications affect chromatin structure and nucleosome dynamics, impacting DNA repair, replication, stability, and cell state transitions. Histone methyltransferases mark methyl groups in the genome, whereas histone demethylases are responsible for removing these marks. These interactions affect chromatin structure and gene expression, thereby regulating gene activity or silencing states (71). Cellular homeostasis and the onset of tumors are decisively influenced by epigenetic and genetic changes (72). Furthermore, lncRNAs play a crucial role in tumor formation by precisely regulating complex control mechanisms. Epigenetic markers vary across different cancers in females, but they affect physiological processes. Currently employed epigenetic technologies such as transcriptomics, through methods such as RNA bisulfite sequencing and Aza-IP, facilitate the detection of cytosine methylation in RNA, aiding genome sequencing. Current epigenetic-based treatments for cancers in females, such as CRISPR and the DepMap database, identify cancer cell vulnerabilities and provide synthetic guidance for targeted drugs, effectively treating female patients. Clinical studies have reported that epigenetic drugs play a significant role in *in vitro* experiments and clinical treatments. More types of these drugs will be produced and used for the treatment of patients with tumors in the future.

The history of cancer treatment dates back several centuries, with many early methods still in use today. Over the past sixty years, significant progress has been made in establishing cancer models and precision therapies. For instance, genomic testing technologies have assisted doctors in tailoring treatment plans based on the characteristics of a patient’s tumor, significantly improving the efficacy of targeted therapies. Epigenetic regulation plays a crucial role in cancer treatment: it enhances the effectiveness of targeted drugs by influencing gene expression in cancer cells. Furthermore, cancer cells often evade treatment through epigenetic alterations, and drugs targeting these changes (such as histone deacetylase inhibitors) can restore cellular sensitivity to chemotherapy and targeted therapies, overcoming drug resistance. Additionally, epigenetic analyses can identify specific biomarkers that aid in developing personalized treatment plans. Moreover, epigenetic regulation can also affect the immune response in the tumor microenvironment, enhancing the attacking capacity of immune cells and improving the efficacy of immunotherapy (191).

### Current limitations in research

Next-generation sequencing (NGS) technologies comprise a series of advanced genomic sequencing methods that allow for high-throughput, rapid, and cost-effective sequencing of DNA and RNA. The advancement of this technology enables researchers to obtain gene expression data at the single-cell level, overcoming the limitations of traditional large-scale sequencing methods in cancer research. Through single-cell transcriptomics and spatial transcriptomics, researchers can more accurately pinpoint gene expression in specific cell types or tumor locations, laying the groundwork for precision therapy. However, current single-cell and spatial transcriptomics technologies still have limitations in resolution, only detecting highly expressed genes, which poses a significant challenge for detecting low-expressed genes. The application of next-generation sequencing technologies can reveal cell-specific gene activity, helping researchers gain deeper insights into the biological mechanisms of cancer, thereby reducing the risk of ineffective treatments. Furthermore, integrating transcriptomics, proteomics, and computational models will provide a more comprehensive perspective on gene regulation, driving breakthroughs in cancer therapy (192).

There are still many limitations to epigenetic research, particularly regarding technical constraints. For instance, the sodium bisulfite conversion method used for DNA methylation testing presents challenges. Sodium bisulfite-treated DNA templates are unstable and prone to degradation, which can lead to difficulties and errors during PCR amplification (193). To address this issue, scientists have developed alternative methods for methylation sequencing analysis, such as methyl-sensitive restriction enzyme (MRE) digestion and methylated DNA immunoprecipitation (MeDIP), followed by high-throughput sequencing (194). In MRE digestion and MeDIP methods, methylated DNA regions are subjected to specific restriction enzyme digestion or affinity enrichment and then analyzed by high-throughput sequencing. However, these techniques often yield low-resolution and limited genomic coverage results and may not effectively differentiate individual methylation backgrounds (195). Furthermore, some epigenetic modifications exhibit high dynamism and cellular heterogeneity, underscoring the need for technical advancements. From a data interpretation perspective, the data generated by epigenetic studies are vast and complex and require precise analysis and interpretation. Uncertainties regarding the function and significance of some modifications remain, and the heterogeneity of patient samples adds complexity to data interpretation. Using technologies such as CRISPR also requires a larger patient cohort to enhance data interpretation. Currently, Cas9 and Cas12a are the only CRISPR family members used for genome editing; however, research is underway to develop artificial variants of these proteins to recognize different protospacer adjacent motifs and target a broader range of genomic sequences (196). Therefore, gene editing technologies need to be applied more widely. While many epigenetic modifications and transcription factors, such as those involved in WNT signaling and DKK3 promoter methylation, have been identified, the functions of many others remain unknown. For instance, whether KLF14 influences macrophage immune function through glycolysis remains unknown (197). A deeper understanding of the normal and abnormal functions of these modifications is essential and requires further research. Despite these challenges and limitations, continued technological advancements and in-depth research are expected to resolve these issues and enhance our understanding of the significant role of epigenetics in biology and disease development.

### Prospects for future research and its significance

Future research into the mechanisms of female cancer development and the impact of epigenetics on female cancers will become more profound. Currently, DNA methylation and histone acetylation are among the most studied epigenetic changes in cancer progression and drug resistance. For example, in estrogen receptor-positive (ER+) preclinical models, small molecule inhibitors have been explored, including histone deacetylase inhibitors (such as entinostat and vorinostat) and DNA hypomethylating compounds (such as decitabine and 5-azacytidine). These compounds are being studied as resensitizers for endocrine therapy (198) (199). Regarding uterine fibroids, additional findings suggest that MED12 mutations are true drivers of fibrotic transformation. MED12 can activate CycC-CDK8 in the kinase module, and its mechanism involves the direct binding of MED12 to CDK8. This binding relies on repeatedly mutated MED12 residues in uterine fibroids (200), which may alter the T-loop conformation and impair CDK8’s kinase activity. Studies have shown that pathogenic mutations in exon 2 of MED12 can disrupt CDK8/19 kinase activity in patients with uterine fibroids (200), revealing molecular defects associated with fibroids. Although MED12 can also regulate transcription independently of CDK8, its mutations are linked to multiple pathology-related signaling pathways (such as Wnt/β-catenin and AKT/mTOR), and future research will focus on their effects and regulatory mechanisms on these pathways (201).Additionally, recent research findings indicate that Cobimetinib exhibits effective anti-cervical cancer activity in multiple cell lines, and when used in combination with paclitaxel, it can synergistically inhibit the growth of cervical cancer cells. This inhibitory effect is achieved by suppressing the activation of the MAPK/ERK signaling pathway while inducing caspase-dependent apoptosis. Furthermore, paclitaxel activates ERK in cervical cancer cells, and this activation can be reversed by cobimetinib (202). Theoretically, it is hoped that the impact mechanisms of epigenetics on cancer development can be more clearly studied in the future, providing theoretical support for technological development. In terms of technology, gene knockout technologies like CRISPR and cell analysis technologies like OriPRINT should receive more attention and development, enabling every female cancer patient to receive targeted treatment and improve survival rates. Clinically, future research could conduct more clinical experiments and treatments on a larger number of female patients based on the safety of animal experiments and promote the development and application of more drugs related to epigenetic factors. In the future, it is hoped that female cancer patients will not only receive effective treatment but also see improvements in quality of life and life expectancy post-treatment. QALY is also an issue that future research institutions need to consider, where QALY principles combine life duration (mortality) and quality of life (morbidity) into a single standard of measurement (203). This combination allows for the comparison of various interventions in the healthcare sector (204). This study can provide new insights into the treatment of female cancers, offering new hope to female cancer patients with diverse treatment options. Starting from the basic principles of epigenetics, to the application of transcriptomics in cancer treatment, and finally to clinical practice, this study helps to uncover innovations in epigenetic approaches to female cancer treatments. Through studies on DNA methylation, histone sequencing, RNA sequencing, CHIP-seq, and other methods, the importance of epigenetics in female cancers has been demonstrated, promising further advancements in research on female cancers.

### Future directions

In the field of female oncology, epigenetics is crucial for the development of early diagnosis and treatment strategies (205). DNA methylation and histone modifications, as key epigenetic markers, play an important role in tumor development. Additionally, non-coding RNAs such as miRNA and lncRNA also play key roles in regulating gene expression (206) and have the potential to become new biomarkers or therapeutic targets. Changes in chromatin structure within tumor cells are also critical; studies on chromatin remodeling help deepen our understanding of the molecular mechanisms of cancer and may reveal new treatment strategies. Meanwhile, liquid biopsy technologies (207), by analyzing circulating tumor DNA (ctDNA) and RNA in blood (208) or other body fluids, provide a non-invasive method to detect tumors, monitor disease progression, and assess treatment effectiveness, demonstrating their huge potential in clinical applications. The comprehensive application of these technologies offers multiple avenues for further research into female cancers (209). Epigenetic mechanisms play a complex role in treating female-specific cancers like breast and ovarian cancer, including DNA methylation and histone modifications (210). A major challenge is how to precisely identify and target these modifications to effectively restore normal gene expression. There are also research gaps in the field of early detection of breast cancer. Although liquid biopsy has made progress in monitoring treatment efficacy and tumor resistance, it is still in the exploratory phase for early detection, particularly lacking comprehensive testing panels for specific gene methylation. Additionally, current studies often focus on single biomarkers (such as CDH1 and RASSF1) and have not established effective integrated gene panels that combine multiple biomarkers to improve detection sensitivity and specificity (211). Additionally, the epigenetic characteristics of cancer can change over time, affecting the progression of the disease and treatment response; how to track and utilize these dynamic changes for real-time assessment is also an urgent issue to address. At the same time, the variability in epigenetic characteristics among patients poses challenges to achieving personalized treatment plans, requiring precise genomic analysis to predict treatment responses and devise corresponding treatment plans (212). Based on these challenges, in the future, we can seek and validate new biomarkers that help predict patients’ responses to DNA methylation inhibitors, thereby improving treatment success rates. We can utilize technologies like liquid biopsies to dynamically monitor the epigenetic changes in cancer patients, adjust treatment plans in real time, and enhance the accuracy of treatment effects and prognosis predictions.

## Glossary

DNA: Deoxyribonucleic Acid
PD-L1: Programmed Death-ligand 1
HLA: Human Leukocyte Antigen
TCR: T Cell Receptor
EZH2: Enhancer Of Zeste Homolog 2
NOL6: Nucleolar Protein 6
TWIST1: Twist-related Protein 1
CpG: Cytosine-Phosphate-Guanine
CGIs: CpG islands
DNMTs: DNA methyltransferases
TET: Ten-eleven translocation
5mC: 5-methylcytosine
TDG: thymine DNA glycosylase
PTMs: post-translational modifications
ADP: Adenosine Diphosphate
FAK: focal adhesion kinase
HDACs: histone deacetylases
siRNAs: Small Interfering RNAs
miRNAs: MicroRNAs
piRNAs: Piwi-interacting RNAs
AGO: Argonaute
PIWI: P-element-induced Wimpy Testis
tsRNAs: tRNA-derived small RNAs
SK-OV-3: Ovary Adenocarcinoma 3
HMBOX1: Homeobox Containing 1
5S rRNA: 5S Ribosomal RNA
tRNA: Transfer RNA
HPV16: Human Papillomavirus Type 16
WNT: Wingless-type
DKK: Dickkopf
ERα: Estrogen Receptor alpha
IDH: Isocitrate Dehydrogenase
CTCF: CCCTC-binding Factor
SMARCA: SNF-related, Matrix-associated, Actin-dependent Regulator of Chromatin 2/4
ARID1A: AT-rich Interactive Domain-containing Protein 1A
ASCUS: Atypical Squamous Cells of Undetermined Significance
CIN2: Cervical Intraepithelial Neoplasia Grade 2
PITX2: Pituitary Homeobox 2
QIAGEN: QIAamp Cador Pathogen Mini Kit
CE: Capillary Electrophoresis
Aza-IP: Aza-immunoprecipitation
scRNA-seq: Single-cell RNA sequencing
CRISPR: Clustered Regularly Interspaced Short Palindromic Repeats
Smart-seq: Switching mechanism at 5’end of the RNA transcript
EMSA: Electrophoretic Mobility Shift Assay
DNase I: Deoxyribonuclease I
CUT&RUN: Cleavage Under Targets and Release Using Nuclease
CUT&Tag: Cleavage Under Targets and Tagmentation
BAX: Bcl-2-associated X Protein
BAK: BCL2 antagonist/killer
MRE: Methyl-sensitive Restriction Enzyme
MeDIP: Methylated DNA Immunoprecipitation
