# Supplementary material for: Transcriptomic era of cancers in females: new epigenetic perspectives and therapeutic prospects
Source: Front Oncol. 2024 Nov 13;14:1464125. doi: 10.3389/fonc.2024.1464125 (PMC11598703; doi:10.3389/fonc.2024.1464125)
Supplement: Supplementary file 1 [file Table1.docx]

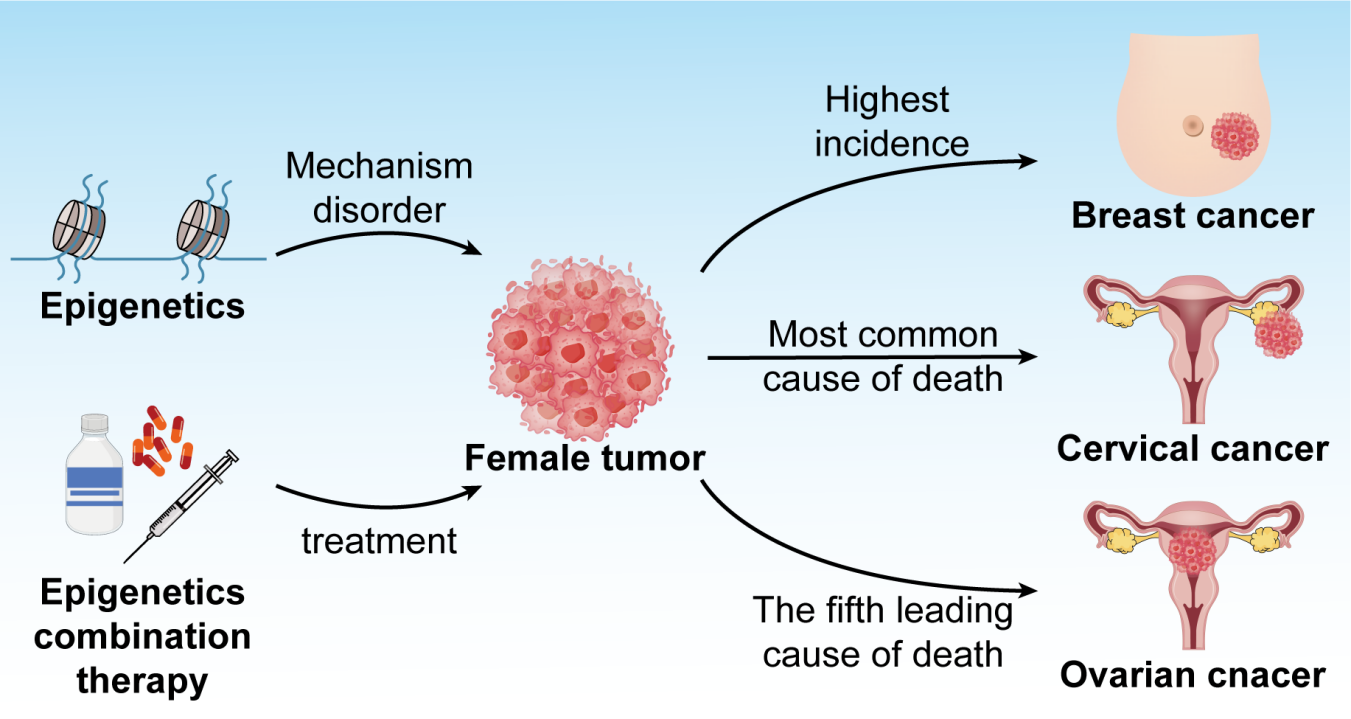


Epigenetics is increasingly cross-developing with oncology in modern times. Epigenetic mechanism disorders can lead to breast cancer, cervical cancer, ovarian cancer and other female tumors. Epigenetic combination therapy has become a new research direction in the development of epigenetics in oncology.
